# Neuromorphological Atlas of Human Prenatal Brain Development: White Paper

**DOI:** 10.3390/life13051182

**Published:** 2023-05-13

**Authors:** Alexandra Proshchina, Anastasia Kharlamova, Yuliya Krivova, Olga Godovalova, Dmitriy Otlyga, Victoria Gulimova, Ekaterina Otlyga, Olga Junemann, Gleb Sonin, Sergey Saveliev

**Affiliations:** Avtsyn Research Institute of Human Morphology of Federal State Budgetary Scientific Institution “Petrovsky National Research Centre of Surgery”, Tsurupi Street, 3, 117418 Moscow, Russia; grossulyar@gmail.com (A.K.); homulkina@rambler.ru (Y.K.); asinello@gmail.com (O.G.); otlyga@bk.ru (D.O.); gulimova@yandex.ru (V.G.); tsvetkovakatya@mail.ru (E.O.); ojunemann@yandex.com (O.J.); glebs0nin@yandex.ru (G.S.); embrains@mail.ru (S.S.)

**Keywords:** human brain atlas, human prenatal development, fetal brain, proteome, forebrain, neurogenesis

## Abstract

Recent morphological data on human brain development are quite fragmentary. However, they are highly requested for a number of medical practices, educational programs, and fundamental research in the fields of embryology, cytology and histology, neurology, physiology, path anatomy, neonatology, and others. This paper provides the initial information on the new online Human Prenatal Brain Development Atlas (HBDA). The Atlas will start with forebrain annotated hemisphere maps, based on human fetal brain serial sections at the different stages of prenatal ontogenesis. Spatiotemporal changes in the regional-specific immunophenotype profiles will also be demonstrated on virtual serial sections. The HBDA can serve as a reference database for the neurological research, which provides opportunity to compare the data obtained by noninvasive techniques, such as neurosonography, X-ray computed tomography and magnetic resonance imaging, functional magnetic resonance imaging, 3D high-resolution phase-contrast computed tomography visualization techniques, as well as spatial transcriptomics data. It could also become a database for the qualitative and quantitative analysis of individual variability in the human brain. Systemized data on the mechanisms and pathways of prenatal human glio- and neurogenesis could also contribute to the search for new therapy methods for a large spectrum of neurological pathologies, including neurodegenerative and cancer diseases. The preliminary data are now accessible on the special HBDA website.

## 1. Introduction

Human brain atlases have been growing rapidly, driven by the increasing availability of modern methods for image visualization and digitization of new and already existing datasets [1,2,3]. As the 21st century is considered the century of the brain, several large-scale brain research projects have been launched: The BRAIN Initiative (USA), Human Brain Project (HBP) (EU), The China Brain Project, The Brain/MINDS (Japan). Israel, Canada, Australia, and South Korea have their own national brain research programs. There are also several international [4] and private initiatives [5].

Brain atlases make it possible to define the organization of brain structures, their shape, relative localization, etc. [6,7]. They are widely used in experimental neuroscience and medicine as tools for locating and targeting specific brain structures [2,3,8], and in education as useful online handbooks [9].

The history of human brain atlas development is reviewed in many papers [2,6,7,8]. In brief, the early architectonic mapping of the human brain was advanced at the beginning of the 20th century, including architectonic areas by Campbell [10], Smith [11], and Ekonomo and Koskinas [12]. The most well-known classic architectonic map of the human cerebral cortex was proposed by Brodmann [13]—a successor of Vogt, who also developed neuroanatomy within the field of brain function localization [14,15]. The earliest atlases of the human brain were derived from one, or at best a few, individual post mortem specimens [7], or even from fragmentary cortical preparations, as in the case of the Economo–Koskinas map, but these maps were created from the detailed histological examination of big brain samples and pathological data, including those of brain injury patients. One of the most complete and definitive brain maps is the Cytoarchitecture Atlas of the Human Cerebral Cortex provided in 1955 [16] by the Brain Institute, Moscow, which was founded by Vogt.

The first digitized brain atlas was developed in the 1970s. It was a natural step forward in brain atlasing, making it possible to overcome the limited functionality of printed books. Recently developed neurobiology methods make it possible to construct spatial brain maps and atlases extending from gross morphology to tissue and cell resolution, including specific transcriptional maps [2,8].

Atlases of the developing brain should be considered separately. Brain growth and maturation are highly complicated processes. Neurodevelopment determined by morphogenetic, genetic, and environmental forces shapes brain morphology, neural architecture, and synaptic circuitry [17]. Cortex development studies can also provide insight into the participation of neurons and brain circuits in human emotion, behavior cognition, and learning. Guides and atlases on neurodevelopment brain anatomy are needed for neuroscientists and clinicians in research and medical practice [18]. 

Current developmental biology contains a variety of data on the differentiation of stem and embryonic cells, intercellular interactions with the definition of molecular mechanisms of auto-and paracrine regulation, transcriptome features of differentiated cell lineages, and other information at the intersection of embryology, molecular biology, and genetics. The majority of such studies are performed on model animals, cell cultures, and living sections. However, data from model experiments cannot be directly extrapolated on humans and need validation [19]. The development of the central nervous system differs in rodents—the most popular model animals—and humans from the early embryonic stages of neuronal tube closure. Moreover, the mouse brain is lissencephalic, i.e., there are no multiform gyri and sulci; thus, one of the main questions of human developmental neurology could not be resolved in the rodent models. The brain maturation dynamics and timeline are also necessary to define for certain species [20]. 

Evaluation of normal human neurogenesis is necessary to understand the mechanisms of regenerative potential in postnatal ontogenesis. The underlying causes of many brain disorders are still not well understood at cellular and tissue levels. Moreover, a lot of psychiatric and neurological pathologies have prenatal origins and cannot be adequately studied in model experiments [17,21]. In addition, organogenesis studies are important for obstetrics and perinatology due to morphological-functional criteria that underlie newborn health. 

The majority of modern publications on normal and pathological human brain development are the data obtained using noninvasive techniques, mainly magnetic resonance imaging (MRI) and its modifications—functional MRI and diffusion tensor imaging [22,23,24,25,26,27,28,29,30,31,32,33,34,35,36,37]. The majority of atlases of the developing human brain, including practical medical atlases, are also based on such data [18,38,39,40,41,42]. In utero MRI provides anatomical details of the fetal brain within a millimeter resolution; thus, it is an important tool for prenatal screening, complementary to ultrasound examination, which gives stronger evidence for identifying gyrification abnormalities, corpus callosum dysgenesis [43], and abnormal cortical maturation. Moreover, a quantitative analysis of in utero MRI images improves the understanding of normal fetal brain development and individual variations (for review, see [18,44]). 

Unfortunately, the resolution of noninvasive techniques is still insufficient for the study of human brain development at the level below macromorphological, and the correlation between such data and real anatomical and histological characteristics of the developing brain still remains a separate field of research [45,46,47,48]. Additionally, standard diffusion tensor atlases lack information in regions of fibre crossing and are based on adult anatomy. The degree of error associated with applying these atlases to studies of children, for example, has not yet been estimated but may lead to suboptimal results [49].

Therefore, the development of fetal brain atlases is limited compared to that of neonatal, paediatric, and adult brain atlases [3,44]. The development of such sources is related to human fetal brain autopsy examination and thus is limited by objective and ethical reasons. In addition, human fetal autopsy samples are especially difficult material and require demanding protocols and procedures, restricted by the legislation and local medical practice. Experimental studies are not principally possible. However, normative fetal brain templates or atlases at matching gestational ages are essential for both research projects and medical practice. 

Several studies have provided the human brain transcriptome profile with an expression of developmental dynamics (for review, see [50]). Even novel technologies, which are based on single-cell sequencing for the spatial gene expression analysis on sections of certain brain regions and structures, cannot fill in the basic knowledge gaps in specific human brain neurology. The technology used for a single-cell RNA-seq analysis, and the spatial transcriptome analysis for sections, has quite strict limitations, such as material requirements (fresh samples frozen in accordance with protocols); furthermore, the results of this method are not sufficient for mapping single neuroblasts. 

Therefore, a histological and immunohistochemical examination is currently the only technique that actually maps the brain directly [51].

Studies on human brain development at the level below macromorphological are mainly related to the names of researchers developing PaskoRakic’s ideas, and/or specifically dedicated to the problem of postnatal neurogenesis [52,53,54,55,56]. Modern studies are concentrated on the molecular and tissue mechanisms of the migration and differentiation of neuroblasts. Mechanisms of the formation of the cortical plate are studied in detail both in model experiments and in the primate neocortex [57,58,59,60] and paleocortex [61,62,63,64,65]. However, even when studies are performed directly on human brain autopsies, they are concentrated on general issues and molecular genetics mechanisms of neuroblast migration [52,53,66,67,68] or related to the problem of postnatal ontogenesis, where human fetal material is used only for comparison purposes [54]. There are only a few works describing developmental translational dynamics of certain human brain regions (cerebral cortex [69,70]; cerebellum [71]; hippocampal region [72]; olfactory bulbs [73,74]; telencephalon [75,76,77]; prepiriform cortex [78]). Such studies are rare and not systemized. They are also still based on separate antigen examinations: immunohistochemical typing of nervous system cells with certain markers remains one of the most important techniques of neurobiology, including research programs on neuropathological processes [20]. The most recent studies are still performed using classic immunohistochemistry with immunoperoxidase and immunofluorescence methods [54]. While some progress has been made in single-cell proteome profiling, it is not currently possible to profile entire proteomes at scale in single cells because of methodological limitations. Instead, antibodies to specific proteins can be multiplexed. Multiplex protein-capture methods can measure protein signatures at the highest resolution, from cellular to single molecules, depending on the technique [79,80].

The integration of newly obtained and existing information remains the main problem: “the gap between the molecular basis of development and already established ideas on the organogenesis, between ultrasonography results and anatomy, data from the dated textbooks and guides on histology and embryology, obstetrics and neonatology, and the current level of understanding of human intrauterine development becomes wider” [81]. This problem is global. In general, the following points concerning the problem should be highlighted:

1. Brain maturation is a complex morphogenetical process. A detailed study of the early development of the human brain is necessary to understand the normal function of the mature human brain and the causes, manifestation, and progression of pathological processes. Currently, there is a great lack in the fundamental knowledge of human developmental neurology. There is no consistent periodization of the development of fetal brain structures or integrative gyrification hypothesis. This deficiency is related to the fact that the majority of studies in the field of neurobiology are designed as model experiments.
2. Studies on human autopsy material are limited due to the complex reasons. Furthermore, intrauterine fetal death often leads to poor condition of the material even for a routine pathology examination. There are also other limitations, both legally established and in the field of individual and professional ethics. Thus, there are relatively few research groups over the world dealing with fetal human material. Only the printed Bayer and Altman Atlas [82,83,84,85] sequentially provides data on human fetal brain development.
3. Most of the current publications on the development of the human brain are based on non-invasive research materials (neurosonography, MRI, and X-ray CT), which provide information mainly at the macromorphological level. Histological research materials cover in more detail the embryonic and pre-fetal periods of development, while data on the fetal period are presented mainly by macro-preparations and schemes [82,83,84,85,86]. Published data on human brain development at different fetal stages are not systematized; they relate to individual aspects of development or individual brain structures and are often controversial.
4. Another consequence of the small number of scientific groups dealing with fetal autopsy material is the lack of research performed at the modern methodological level. Current advanced research is concentrated mainly on the features of transcriptome functioning in the developing brain [3,51,56]. Besides the well-known studies of the group of PaskoRakic and his students and colleagues, which have already become classic, immunohistochemical studies of the proteome of the developing human brain are episodic and unsystematic. At the same time, proteins are the main functional molecules in cells. Gene transcription products do not always result in translation, and, therefore, it is not always possible to judge the functioning of a protein product in a cell on the basis of transcriptional activity. Studying the translational mechanisms in human brain development is a relevant problem in current neurobiology.
5. Worldwide, there is a growing interest in the accumulation of large-scale scientific and reference data into databases available for general use. Morphology, including immunomorphological tissue research, generates large amounts of of digital images, which provide valuable information. However, data are mostly provided only as quantity tables or conclusions and do not fully contribute to publishing data in original research papers. Complete and detailed data are not available to a wide range of experts, including neuroscientists and neurologists.

Thus, a digital database on the immunophenotypical cell profiles in the developing human brain is highly requested. The accumulation of data on human brain development, including data on the immunophenotype of various brain regions, remains an important task that has both fundamental and practical importance. The digital high-resolution histological reference annotated atlas of normal prenatal human brain development is a relevant task for human neurobiology. A separate issue is the creation of a consistent database of cortex brain development, covering all stages of intrauterine development, including the fetal period, which will present materials not only at the macromorphological but also at the histological level, taking into account the regional specific features of the immunohistochemical profile of various cortical territories of the maturing big brain. 

Therefore, the Human Prenatal Brain Development Atlas (HBDA) has been launched, which aims to create spatiotemporal reference maps of the whole fetal brain. The first draft of the HBDA will profile 16 fetal brains at various stages of prenatal development. It will combine histological and immunohistochemical methods. The HBDA could help answer questions regarding the histological brain structure, developmental biology, and cell fate and lineage, including neuro- and gliogenesis. This paper describes in detail a research strategy for the HBDA.

## 2. Materials and Methods

### 2.1. Tissue Sources and Processing

**Figure 1:**
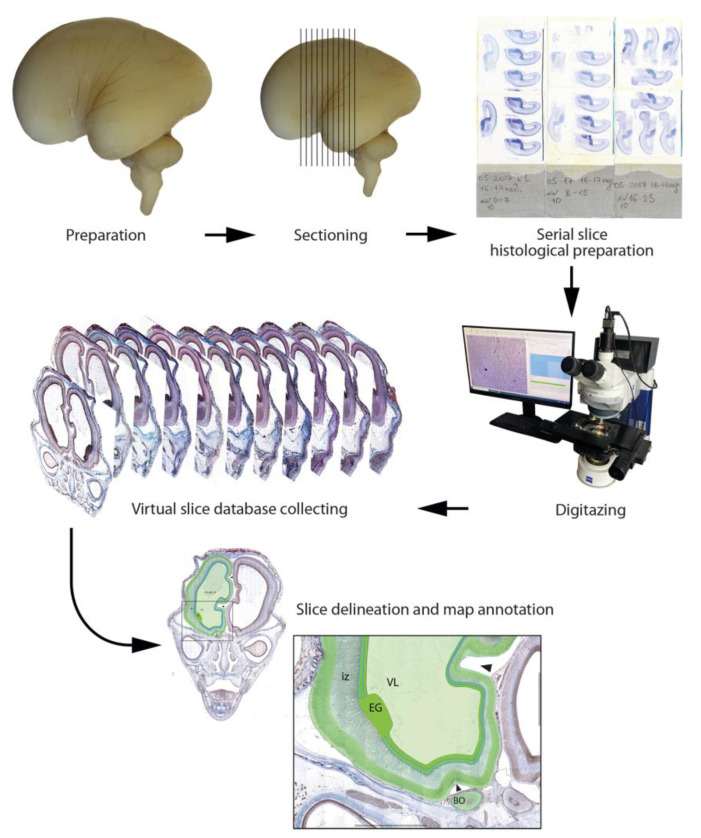
HDBA human fetal brain processing from fixed sample to virtual sections with annotated brain maps.

The HBDA will be based on postmortem human brain autopsies from the unique Collection of the Prenatal Human Development of the Laboratory of the Nervous System Development at the Research Institute of Human Morphology. It consists of the material from the second post-conceptional week (2 pcw) until birth. The materials have been collected in the last five decades and are currently being renewed. The collection consists of more than 200 formalin-fixed human embryos and fetuses at different gestational stages. The collection contains not only fixed material but also histological, immunohistochemical, and electron-microscopic preparations. All the material was taken and handled according to national legislation and the Declaration of Helsinki. Protocols were approved by the local Ethics Committee of the Research Institute of Human Morphology. The classification of the fetal period, which is divided into four stages (pre-fetal (8–12 weeks), early fetal (13–20 weeks), middle fetal (21–28 weeks), and late fetal (29–40 weeks) periods)), will be used [81]. At least four samples from each developmental period will be examined with histological and immunomorphological methods and provided as Atlas specimens at the initial step of the project within the next two years (Figure 1).

### 2.2. Histological Preparation

Most samples are buffered formalin-fixed. For the prenatal reference Atlas, serial coronal and sagittal 5–10 μm thick paraffin sections from the whole embryo or fetal head, fetal brain, or an entire hemisphere, depending on the developmental stage, are stained with hematoxylin-eosin, Mallory trichrome, and Nissl techniques.

### 2.3. Immunohistochemistry (ICH)

**Figure 2:**
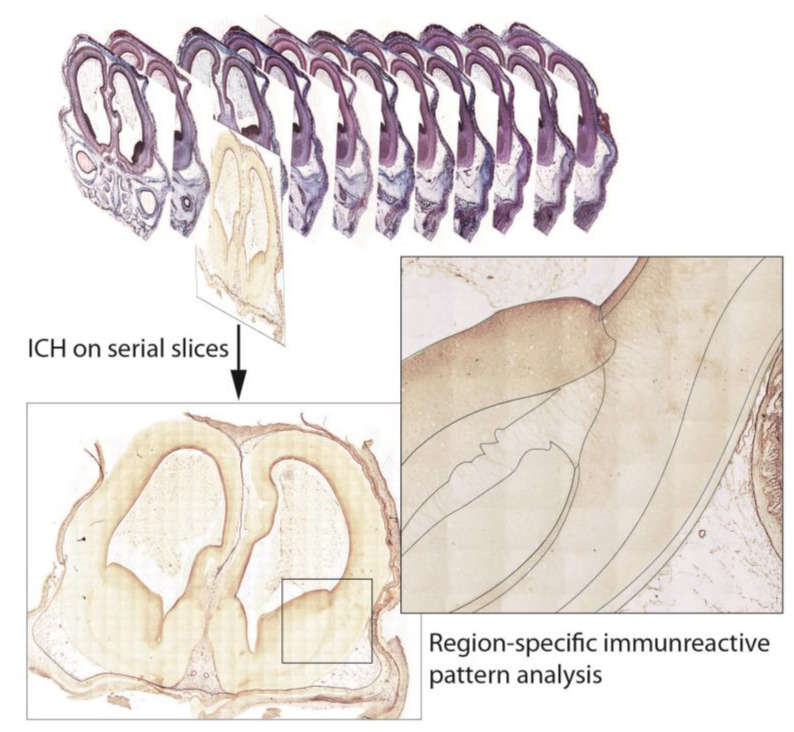
Immunohistochemical reactions on the sections from the serial brain preparation and region specific analysis of the immunoreactive patterns with specific markers.

A combination of the classic cytoarchitecture with recent immunohistochemical methods is a great advantage of the immunomorphological approach that resulted in the regional-specific immunophenotyping of brain areas at various prenatal stages. In accordance with the HBDA objectives, immunohistochemical experiments are planned using direct immunoperoxidase, double, and multiplex labeling with immunofluorescence and chromogen methods directly on a series of histological sections, followed by an analysis using morphometry. After the reference histological application, immunohistochemical experiments will be applied (Figure 2). 

Multiplex immunolabeling will be used for the spatial analysis of antigen patterns on the same slice. Thus, immunophenotypes of the whole brain structure will be obtained as a result of the procedure, and the spatial-temporal rates of the structure maturation will be accessible for examination using morphometry and statistical analysis. 

The mechanisms and factors of the differentiation between multipotent and oligopotent progenitor cells of the nervous system are central problems in recent developmental neurobiology. A series of immunohistochemical experiments with a wide panel of specific markers will be taken to mark the main central nervous system cell populations on the whole fetal hemisphere slice. The choice of a marker panel for the project is based not only on the authors’ own experience but also on the published data and online materials on related sources, such as The Human Protein Atlas, Allen Brain Atlas, and others. Preference in each phase of experimentation will be given to markers that are most well-established in world research and medical practice and consistently work on human fetal autopsy material. Thus, the HBDA will be useful for the comparative analysis with other research groups’ data (neocortex [69,75,87,88,89], archaecortex: hippocampus and dentate gyrus [54,72]). Well-proven markers will be used, including neuronal nuclear antigen (NeuN), glial fibrillary acidic protein (GFAP), and aldehyde dehydrogenase 1 family member L1 (ALDH1L1) for astrocyte lineage typing, the myelin basic protein (MBP) for mature oligodendroglia, vimentin for the identification of radial glia processes at the pre- and early fetal stages of development, and others.

Additionally, markers of the immature cells such as anti-DCX and anti-Ki-67 will be used. The nuclear protein Ki-67 is expressed in the interphase and mitotic phase of the cell cycle (absent in G0) [90,91]. This protein is widely used in studies of proliferation and neurogenesis not only in tumors but also during normal development [72] and adult neurogenesis [92]. DCX is the protein associated with the microtubules of the cytoskeleton and is synthesized predominantly in immature neurons [93]. However, DCX expression and synthesis persist in some regions of the adult mammalian brain [94,95,96]. The DCX-positive cells in some cortical regions of the adult brain are suggested to be related to the presence of immature cells preserved from the prenatal period of active neurogenesis. However, some authors have reported that the DCX-positive cells in the adult human cortex are not immature neurons [97]. In any case, DCX as a cytoskeleton-associated antigen is a promising marker for human autopsies, because cytoskeleton markers are less demanding (in comparison to PSA-NCAM, for example) to the time of fixation and the degree of preservation of the material than antibodies to nuclear or surface membrane antigens.

A series of immunohistochemical experiments will be carried out in order to characterize neuroblasts of the neuronal and glial differentiation lineages. The application of antibodies against transcriptional factors associated with one of the cell differentiation lineages—neuronal, astrocyte, oligodendrocyte—is widely used in recent developmental neurobiology [54,55]. Antibodies to transcriptional factors allow typing of different neuroblast lineages in the early stages of maturation, before the appearance of cytological features or proteins that are specific for mature cells. The Olig family is a perspective transcriptional factor for typing early oligodendrocytes [98,99]. Anti-Olig-2 will be used for the typing of early oligodendroblasts. To clarify the role of other transcriptional factors (-Sox9, -Ascl, -ARX, -Tbr1, -Hes5/7) and proteins (FABP7), which are believed to participate in central nervous system development and the differentiation and maturation of cell lineages, experiments will be also undertaken.

The functional markers may be used as indirect indicators of the functional activity in the process of cell maturation in nervous tissue. Antibodies to the functional proteins of presynaptic terminals, including synaptophysin, are classically used for the monitoring of synaptogenesis and functional maturation of neural tissue [87,100,101,102]. Expression of another functional protein, neuron-specific enolase (NSE), begins immediately after the formation of neuronal afferents and efferents, and the distribution of NSE also correlates with the maturation of neural tissue [103,104]. Analysis of the distribution of the main central system mediators is also planned.

### 2.4. Human Brain Imaging

Annotated atlases based on the series of virtual slices of sixteen human fetal brains in different developmental stages will be a main component of the HBDA.

The basic annotated atlases will contain an image library of virtual sections with a zoom tool corresponding to an objective resolution of up to ×20. Virtual slices from the serial histological preparations of human fetal brain autopsies will be made using the MECOS-C2 system, which is specially modified for large (non-standard) preparation sizes (up to 12 × 9 cm).The system collects hundreds of images in lengthwise strips, which are later stitched together to create one large image. The exposure time, white balance, and flat-field correction are set independently for each slide. The acquired SVS files will subsequently be converted to JPEG. As the initial step of the project, histological preparations from the collection will be digitized, annotated, and analyzed to create a reference base for the Atlas. New preparations will be prepared especially for the HBDA (Figure 2).

The HBDA team already has experience in digitizing preparations and creating a virtual collection, including fetal brain samples. Archival preparations (2600 × 3000 px) from the Laboratory Collection are already available on the special laboratory website(https://brainmicroscopy.com/collection/homo/brain-development/, accessed on 28 March 2023), which is granted by the Historical Committee of the Federation of European Neuroscience Societies (FENS). The HBDA results will be organized as annotated atlases of the developing human brain, with a series of virtual sections, with a resolution of up to 20,000 × 20,000 px; moreover, new results from the fundamental neurodevelopmental study, with regional-specific immunophenotype profiles of the fetal human telencephalon, will be provided, and developmental heterochrony in human cortical development will be highlighted.

### 2.5. Data Organization, Neuroanatomical Parcellation, Annotation of 2-D Sections

**Figure 3:**
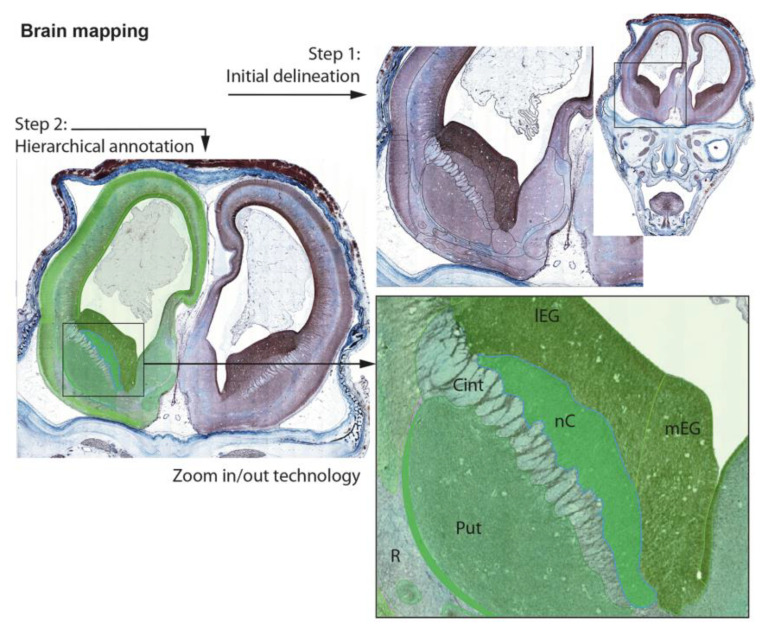
Delineation and mapping of the virtual sections from the digital fetal brain collection.

A separate task is marking and annotating virtual preparations for each developmental stage. Annotation schemes will be overlapped on the digital images of histological slides. The resulting vector graphics will be converted to Scalable Vector Graphics (SVG). Each vector area will then be associated with a certain brain structure and annotated; in this way, the collating vector allows the flexibility to create various presentation modes (e.g., with or without colorization and transparency) (Figure 3).

There is no one uniform nomenclature for all structures of human developing telencephalon. Structural delineations will be based on classic adult human brain morphology and special publications on the certain structure developmental course, with special references for evolutionary nomenclature principles. All published sources used for each structural complex will be provided as special comments. Differences between existing neurologic traditions will be compared and commented on, with special references to classic German, American, and Russian (Soviet Union) ontological principles and the recent “standard” of anatomical ontology provided in Nieuwenhuys et al. [105], Terminologia Neuroanatomica [106] and the Allen Brain Atlas as an online source [107]. The structure will be delineated and annotated following comparative evolutionary and ontogenetic criteria—from the general to the particular, from complex structures to parts and subdivisions according to the developmental course.

## 3. Discussion, Highlights, and Perspectives

The primary goal of the HBDA is to create the immunophenotype neurogenetic map of the developing human telencephalon, which may underlie an analysis of regional heterogeneity and heterochrony under the developmental course of human normal brain maturation. The formation and maturation of the forebrain are among the main topics in recent neurobiology since the human cortex is a functional base for human cognition and intelligence. Studying the prenatal human telencephalon contributes to solving fundamental questions of developmental neuroscience and also greatly develops special human neurology. Within a great number of studies devoted to molecular and genetic mechanisms of neuroblasts migrations, basic themes such as periodization of normal development, ranges and variabilities, comparative functional morphology, and cytoarchitectonic analysis of various regions of the developing human brain remain poorly covered. There are almost no complex studies on the recent methodological level devoted to region-specific normal morphogenesis of the human brain. Published studies within the last decade are very limited and focused on certain regions of the developing brain, and are often performed on limited material (paleocortex: hippocampus, dentate gyrus [54,72]; neocortex, early fetal period [89]; cerebellum, pre- and early fetal period [71]). There are studies devoted to region-specific expression of individual genes specific for neural diseases (NDRG2, the end of early fetal period and middle fetal period) [108]. Several studies describe region-specific expression and synthesis of some transcriptional factors in the human forebrain [76,77]. The role of such region specificity in morphogenesis is not yet fully understood.

The region-specific analysis is the key to understanding morphogenesis and the differentiation of such a complex structure as the human brain. The development of current spatial transcriptome analysis techniques of individual organs and tissues further supports this fact. A modern technique like single-cell sequencing (single-cell RNA-seq analysis), applied with regional specificity in mind, indicates the heterogeneity of, at least, the neuronal differentiation between the large regions of the forebrain, and the existence of regional specificity in the expression (transcription) of genes related to neural diseases between different brain regions [109,110,111]. However, such technologies are still more restricted in morphological analysis compared with classical ICH and in situ hybridization. Despite the recent clinical and scientific advances in radiological diagnostic, multimodal monitoring, laboratory diagnostics, genomics, and proteomics, there is also still a need to identify new biomarkers that can be used for diagnosing brain disorders [112].

The development of CNS malformations involves various processes, including abnormal neurulation, proliferation, migration, etc. [113]. As mentioned above, the resolution of non-invasive methods for studying the developing brain is much lower compared to histological methods. [45,46,47,48]. Thus, comparing data obtained by different methods and distinguishing between various stages of development is crucial for identifying developmental abnormalities. This will enable medical professionals to promptly detect any embryonic complications [113,114].

Undoubtedly, atlases of human brain development still have a long way to go in terms of detailed resolution. For several invertebrate species, including *Caenorhabditis elegans*, the tadpole larva of *Ciona intestinalis*, and two annelid species, the polychaete *Platynereis* and the leech *Hirudo verbena* [115], connectomic data have already been reported. An interactive atlas of the Drosophila nervous system, Virtual Fly Brain, has also recently appeared [115,116]. This problem is not solved for vertebrates, including human, which is associated with much larger brain sizes. However, it is precisely research on the human brain that is the key to understanding problems related to consciousness, thinking, and human behavior as a whole.

With the aim of HBDA, two main steps are needed: immunophenotyping and cell lineage mapping of certain telencephalon subdivisions at various prenatal stages (pre-fetal, early, middle, and late fetal periods) followed by the presentation of project results as a reference annotated online atlas. New data on the developing brain proteome, including regional specifics, will be collected, and the digital source could become a base for further investigation on human brain development.

The human cell proteome contains thousands of proteins. In this project, neuronal and glial cell lineage differentiation will be examined with main specific markers of certain cell populations, including the transcriptional factor and functional neuro-specific markers. The immunomorphological approach makes it possible to compare immunohistochemical cell profiles of different cortical areas with annotated virtual brain slices and region-specifically evaluate the maturation rates of the main cell lineages—astroglial, oligodendroglial, and neuronal—to describe the heterochrony of cortex development and analyze developmental changes and tendencies at the different stages of prenatal human ontogenesis.

The main data of the atlas will be organized as macro-and microphotographs of the embryos and fetuses at different developmental stages, including annotated graphs with morphology and immunohistochemical experiment results, a series of virtual slices implemented with technology for creating digital images for remote viewing without loss of quality and resolution, as well as diagrams superimposed and overlaid with annotated “maps” of fetal brain structures. Systematization and post-processing of the obtained fundamental data involve the creation of diagrams and three-dimensional reconstructions that clearly demonstrate the processes of growth and development of the human brain. In the pilot version of the site, navigation both by the period of development of embryos and fetuses and by individual brain structures or certain key methods and markers is planned.

The developers of HBDA will focus on creating a user-friendly interface with easy switching and cross-references between sections, the hierarchical organization of tabs and links, and convenient switching between them. Navigation through the source is planned separately by the stages of development, histological methods used, immunohistochemical markers, and systems, regions, and structures of the developing human brain.

The Atlas would eliminate the following gaps in the necessary data:

1. A reference database for the developmental research of different human brain structures. These materials are unique, which makes it possible for them to be in demand for many years.
2. Comparative material for general neurology—non-human mammal, cultural, and other experimental studies.
3. A reference database for the comparative analysis of non-invasive (ultrasonography, MRI, CT) study results, newly spatial genomic sequence technology, and high-resolution 3D-visualization of the native tissue samples with phase-contrast CT.

For medicine practice, the results of this project will be useful:

(a) in obstetrics practice—for the comparison of ultrasonography and MRI/CT results;
(b) in neonatology—for brain maturity criteria;
(c) in prenatal and perinatal pathoanatomy—for the intrauterine and neonatal death cause analysis and searching for pathological changes in the fetal brain.

**Figure 4:**
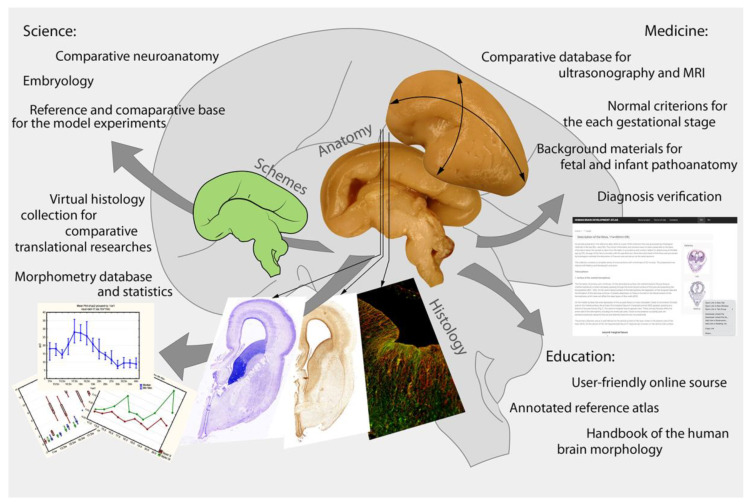
Prospects for using the human prenatal brain development atlas in science, education, and medicine.

To date, many scientists all over the world use such online and offline sources in everyday practice. Such an online source would make it possible to save funding, as well as the time and efforts of specialists and researchers. Therefore, one can expect that the HBDA will be useful as an annotated guidance for fundamental research. An online atlas of the developing brain will also be useful in educational programs on embryology, histology and cytology, neurology, physiology, endocrinology, pathoanatomy, genetics, etc. (Figure 4).

Thus, the HBDA is a research program that connects a fundamental scientific study of the prenatal human brain with a digital presentation of the project and results in an online available renewable resource. The authors believe that the digital atlas of normal human brain development will fully respond to current trends in scientific practice, its methods, and means of communication.

## Data Availability

The anonymized complete dataset will be provided as a basis for further investigations on the special HBDA website at https://brainmorphology.science/ (accessed on 28 March 2023). A part of the Collection samples are also publicly accessible via the laboratory website at https://brainmicroscopy.com/collection/homo/brain-development/ (accessed on 28 March 2023).
